# Transactive DNA Binding Protein 43 Rather Than Other Misfolded Proteins in the Brain is Associated with Islet Amyloid Polypeptide in Pancreas in Aged Subjects with Diabetes Mellitus

**DOI:** 10.3233/JAD-170192

**Published:** 2017-07-03

**Authors:** Marina Leino, Svetlana N. Popova, Irina Alafuzoff

**Affiliations:** a Department of Pathology, Uppsala University Hospital, Sweden; b Department of Immunology, Genetics and Pathology, Uppsala University, Sweden

**Keywords:** α-synuclein, amyloid-β, diabetes mellitus, islet amyloid polypeptide, hyperphosphorylated *τ*, phosphorylated transactive DNA binding protein 43

## Abstract

A link between diabetes mellitus (DM) related islet amyloid polypeptide (IAPP) and Alzheimer’s disease (AD) related amyloid-β (Aβ) has been suggested in epidemiological and clinical studies. In 2017, proof for existing interaction between type 2 DM and AD on a molecular level was provided based on research carried out in experimental animal models. We assessed aging-related neurodegenerative lesions, i.e., misfolded proteins, associated with dementia such as hyperphosphorylated *τ* (HP*τ*), Aβ, α-synuclein (αS), and phosphorylated transactive DNA binding protein 43 (pTDP43) seen in the brain and IAPP seen in the pancreas in subjects with and without DM applying immunohistochemical techniques. HP*τ* in the brain and IAPP in the pancreas were observed in most subjects. The prevalence and the extent of all misfolded proteins increased with age but this increase was not influenced by DM. Interestingly the extent of misfolded proteins in the brain was higher in non-diabetics when compared with diabetics in demented. A significant correlation was observed between HP*τ*, Aβ, αS, and pTDP43, whereas IAPP showed no association with HP*τ*, Aβ, and αS. In subjects with DM, the extent of pTDP43 in brain correlated with the extent of IAPP in pancreas. Thus, there is no evidence of a link between AD-related pathology and DM in humans, whereas an association was found between pTDP43 and IAPP in DM. TDP43 is ubiquitously expressed in all organs but whether TDP43 is phosphorylated in other organs in DM or whether the phosphorylation of TDP43 is influenced by glucose metabolism is yet unknown.

## INTRODUCTION

With aging misfolded proteins, i.e., hyperphosphorylated *τ* (HP*τ*), amyloid-β (Aβ), phosphorylated α-synuclein (αS), and phosphorylated transactive DNA-binding protein 43 (pTDP43) are frequently observed in the human brain tissue [1]. In excess, these proteins are associated with various age related neurodegenerative diseases. In Alzheimer’s disease (AD), HP*τ* and Aβ are deposited in the brain; in dementia with Lewy bodies, αS, and in frontotemporal lobar degeneration, pTDP43 is deposited in various cell compartments [2–5]. The deposition of these proteins is regionally predictable; thus, a reliable assessment of the extent of the pathologies can be carried out [2, 6, 7]. All these proteins can be seen simultaneously in one and the same subject, i.e., mixed pathologies [8, 9].

**Fig.1:**
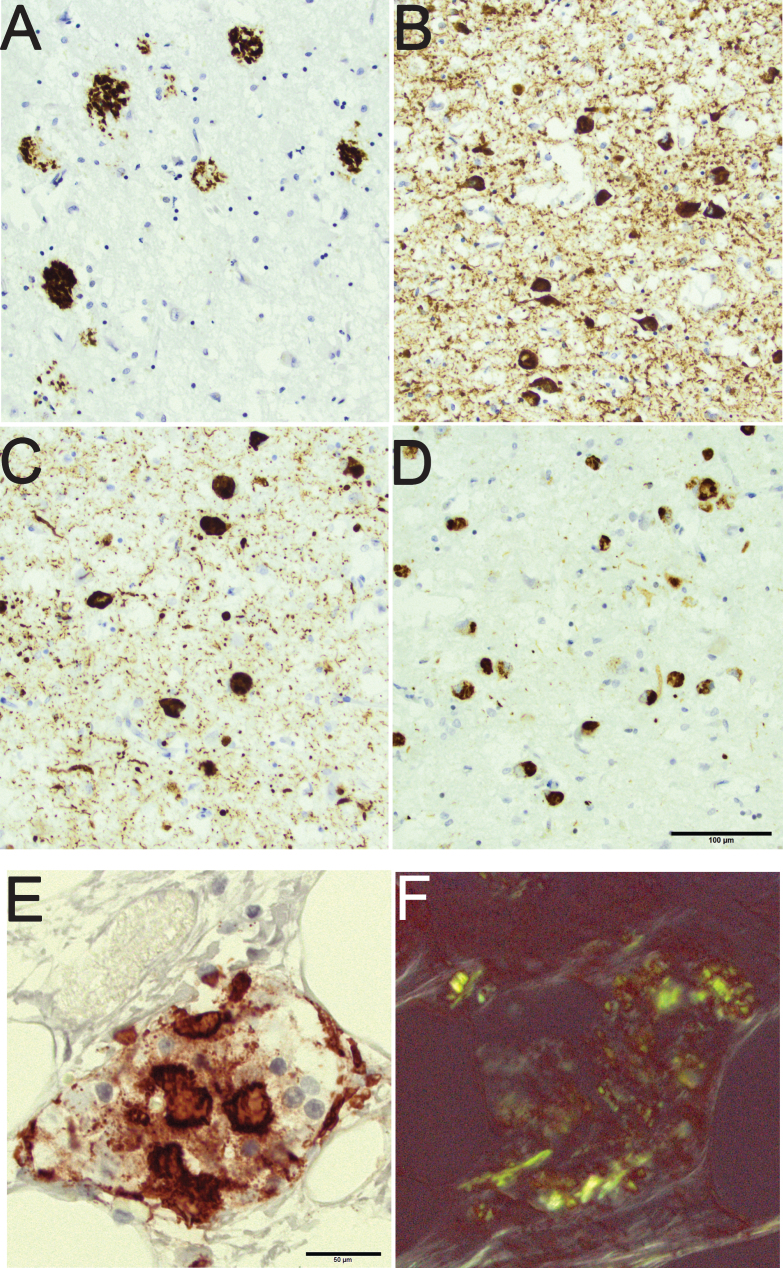
An 80-year-old male with a clinical diagnosis of Alzheimer’s disease with mixed pathology seen in the amygdala. Note the rounded amyloid-β labeled aggregates in the brain parenchyma (A), the hyperphosphorylated-*τ* labeled neurites and intraneuronal tangles (B), the α-synuclein labeled neurites and intraneuronal Lewy bodies (C), and the phosphorylated transactive DNA binding protein 43 labeled cytoplasmic inclusions (D). Pancreas tissue from an 85-year-old female with diabetes mellitus and signs of depression. Note the protein aggregates in an islet of Langerhans labeled with antibody directed to islet amyloid polypeptide (E). The same islet of Langerhans seen in Congo stain (F); note the birefringence of the protein. Scale bar: 100 μm in A-D and 50 μm in E,F.

In diabetes mellitus (DM), deposition of misfolded protein, i.e., islet amyloid polypeptide (IAPP), is observed in the pancreas [10, 11]. IAPP is secreted by the islet cells, and the deposition of IAPP is associated with type 2 DM and in line with AD, type 2 DM is more common in the aged [10, 12]. To our knowledge, there are no reports with human subjects indicating that aging in itself would be associated with deposition of IAPP in the pancreas. Thus, in aging misfolded proteins can be seen in both brain and pancreas as soluble and as insoluble aggregates [13]. The insoluble aggregates have been referred to as “hallmark” lesions of a certain disease and they can readily be visualized as immunoreactivity (IR) as is seen in Fig. 1, in the postmortem tissue, brain and pancreas, applying immunohistochemical (IHC) techniques [2, 6, 7, 14].

Epidemiological studies have shown that there is an association between DM and dementia and thus DM has been suggested being a risk factor for AD [15–17]. It has been suggested that AD related and DM associated “pathologies” might influence each other and that a cross talk and/or cross-feeding might exist between the aggregation prone proteins [18].

In 2017, Moreno-Gonzales and colleagues reported that there is indeed a link between AD and type 2 DM, i.e., misfolded IAPP produced in pancreas in type 2 DM promoted AD pathology (Aβ) by cross-seeding [19]. This was observed both in cell culture and in transgenic animals. Based on the above a question arises as to whether the misfolded protein in the pancreas, i.e., IAPP might influence the protein aggregation in the brain, i.e., βA, HP*τ*, αS, and pTDP43 and vice versa inhumans.

Contrary to the above, neuropathological observations carried out on large human cohorts have not been able to show any link between AD and DM[20, 21]. Moreover, recently in a clinical setting a lack of association was reported between biomarkers of neurodegeneration and type 2 DM [22].

The objective of this study was to investigate further the presumed association between the misfolded proteins in DM and neurodegeneration. We assessed the incidence and the extent of the insoluble aggregated proteins at the end stage, i.e., postmortem. We assessed pathologies such as HP*τ*, Aβ, αS, and pTDP43 observed in the brain tissue and the IAPP observed in the pancreas. The primary objective was to look for if any signs of association, i.e., cross talk and/or cross-seeding between these proteins could be detected. In addition, we looked for signs of colocalization of misfolded proteins in brain andpancreas.

## MATERIAL AND METHODS

### Subjects

**Fig.2:**
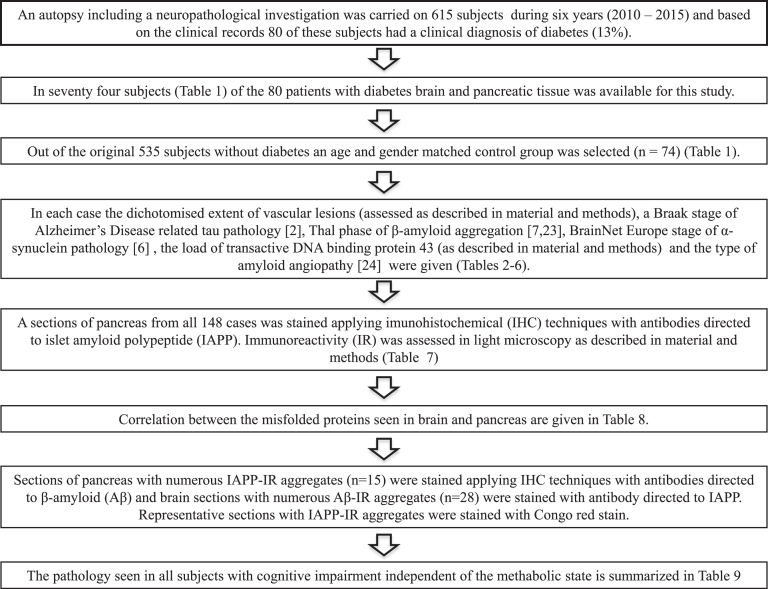
Flowchart.

During a six-year long period (from 2010 to 2015), an autopsy including a neuropathological investigation was carried out on 615 subjects, age ranging from 48 to 102 years, at Uppsala University Hospital (Fig. 2). The medical records revealed that 80 of the deceased subjects (13%) had a clinical diagnosis of DM, age ranging from 48 to 95 years at death. In 74 (age range 48 to 87 years at death) of these 80 subjects, in addition to the brain tissue the pancreatic tissue was available for this study. An age and gender matched non-DM control group, including 74 subjects (49 to 87 years at death), was separated from the available cohort of 535 autopsycases.

All procedures performed in the study involving human participants were in accordance with the ethical standards of the institutional and national research committee. The subjects included and/or their relatives had given their consent for the use of the tissue, and the study has been authorized by the regional Ethics Committee of Uppsala, Sweden # 2011/286. The study was carried out in accordance with the 1964 Helsinki declaration and its amendment or comparable ethical standards.

### Sampling of the tissues

At autopsy, the brains were weighed, fixed in excess of 4% buffered formaldehyde for a few days, and cut into 1-cm-thick coronal slices. Grossly notable lesions were noted as present or not on all fixed coronal slices. Following a standardized protocol, 16 brain specimens were sampled in each case, including areas such as the frontal, temporal, parietal, precentral, occipital cortices, the gyrus cinguli, and the striatum; basal forebrain including the amygdala, thalamus, and the anterior and posterior hippocampus; midbrain including the substantia nigra; pons including the locus coeruleus, medulla, vermis, and the cerebellar cortex.

### Processing of tissue samples

Seven μm thick sections of the paraffin embedded sampled brain tissue (16 blocks/case) was stained with hematoxylin-eosin (HE) stain to assess the microscopic lesions such as infarcts that were noted to be seen or not.

Pancreatic tissue was sampled at autopsy from the body of the organ and fixed in formalin for at least one week. Three μm thick sections of the paraffin embedded material were stained applying HE stain to assess the representability of the tissue.

### Immunohistochemistry

For IHC stains carried out on the brain tissue, 7 μm thick sections and on the pancreas 3 μm thick sections were used. Briefly, HP*τ*/IR was visualized using monoclonal antibody (mAb), clone AT8 (MN1020, ThermoScientific, Waltham, MA) in a dilution of 1:500 without pre-treatment; Aβ/IR was visualized using mAb, clone 6F/3D (M0872, DakoCytomation, Glostrup, Denmark), in a dilution of 1:100 following pretreatment with 80% formic acid for 6 h; phosphorylated αS/IR was visualized using mAb clone KM51 (NCL-L-ASYN, Leica Biosystems Newcastle Ltd, Newcastle upon Tyne, United Kingdom) in a dilution of 1:100 following heat pretreatment (autoclave) in a citrate buffer at pH 6.0 and 80% formic acid for 5 min; pTDP43/IR was visualized using mAb clone 11-9 (TIP-PTD-M01, Cosmo Bio Co. Ltd, Tokyo, Japan) in a dilution of 1:5000 following heat pretreatment (autoclave) in a citrate buffer at pH 6.0; and IAPP/IR was visualized with mAb, clone R10/99 (MA134685, ThermoScientific), in a dilution of 1:200 following heat pretreatment (autoclave) in a citrate buffer at pH 6.0 and 80% formic acid for 5 min. For detection of the antibody binding, the BrightVisonPlus detection system from Immunologic (Duiven, The Netherlands) with Romulin-3-amino-9-ethylcarbazol chromogen (BioCare Medical, Concord, CA) was used.

A representative section of the pancreas with notable IAPP/IR was stained with Congo-red stain to confirm the apple-green birefringence under polarized light (Fig. 1).

### Assessment of stained sections

All assessments were carried out in light microscopy, in magnifications x40–x400. The brain sections to be stained with the antibodies listed above were selected based on the requirements of the recommended staging protocols. For HP*τ*, the Braak stage (0, a,b, I–VI) [2]; for Aβ, the Thal phase (0–5) [7, 23]; and for αS, the Brain Net Europe stage (0–6) [6] were given. The type 1 or type 2 cerebral amyloid angiopathy was assigned as recommended by Thal and colleagues [24]. The pTDP43/IR was looked for and assessed in a dichotomized manner as seen or not seen in the section of the amygdala, medulla, and the hippocampus. All lesion types, i.e., intracytoplasmic inclusion and neurites were assessed. The semi-quantitative extent of pTDP43 was given as (0) not seen, (1) seen in one region, (2) seen in any two regions, and (3) seen in all three regions. The IAPP/IR was assessed by counting the IAPP/IR patches in ten randomly selected fields in magnification x100. A mean value of IAPP/IR was calculated for each case, i.e., sum of IR patches divided by the number of assessed fields.

The IAPP/IR was also sought after on a section obtained from the cortex. Cases that displayed a high load of Aβ (*n* = 28) in the brain were chosen for this purpose. The Aβ was also looked for on a section obtained from the pancreas. Cases that showed a high load of IAPP/IR (*n* = 15) were selected for this purpose.

### Statistical methods

The IBM SPSS statistics software (IBM Corporation, Armonk, NY) was used. Statistical differences were analyzed using non-parametric Mann-Whitney U-test when two cohorts and Kruskall-Wallis test when more than 2 cohorts were compared, and the correlation was assessed by Spearman’s correlation test.

## RESULTS

### Demographics of the subjects

The age at death of the selected study cohort, including 74 subjects with DM and 74 non-diabetics ranged from 48 to 87 years. Out of this cohort 16% had displayed cognitive impairment during life. The brain weight ranged from 988 to 1810 grams and the post mortem delay ranged from 10 to 336 hours. Demographics of the included subjects are given in Tables 1 and 2.

**Table 1:** Demographics of the included subjects

Systemic disease	Age	Mean	Number	Gender	With cognitive	PMD hours
	Groups	Age±SE		F/M	impairment	mean±SE
Non-diabetics	≤65	59.2±1.6	16	8/8	0	107±12
	66–76	71,6±0.6	26	13/13	3	100±11
	≥77	81.8±0.5	32	13/19	11	126±14
	49–87	73.3±1.1	74	34/40	14	113±8
With diabetes	≤ 65	58.6±1.6	15	8/7	1	133±16
	66–76	71.4±0.6	28	14/14	5	100±9
	≥77	81.9±0.5	31	13/18	4	102±10
	48–87	73.2±1.1	74	35/39	10	108±6
All subjects	≤65	58.9±1.1	31	16/15	1	120±10
	66–76	71.5±0.4	54	27/27	8	100±7
	≥77	81.8±0.3	63	26/37	15	114±8
	48–87	73.3±0.8	148	69/79	24	110±5

SE, standard error of means; F, female; M, male; PMD, postmortem delay.

**Table 2:** Altered proteins and vascular alterations in the brain and islet amyloid polypeptide (IAPP) in the pancreas

Systemic	Age	*n*	Brain weight	HP*τ*	Aβ	CAA	αS	TDP43	micro/macro	IAPP IR	IAPP IR^a^
disease	groups		gram±SE	*n* (%)	*n* (%)	Type1/2	*n* (%)	*n* (%)	infarcts	*n* (%)	mean±SE
Non-diabetics	≤65	16	1472±39^1,6^	12 (75)^d^	2 (12)	1/0	1 (6)	5 (31)	2/3	15 (94)	3.3±0.5
	66–76	26	1316±26^1,6^	26 (100)	12 (46)	1/4	3 (12)^b^	13 (50)	2/6	25 (96)	3.7±0.5
	≥77	32	1382±25^1^	32 (100)^c,e^	17 (53)	1/7	9 (28)	19 (59)	1/10	32 (100)	4.1±0.6
	49–87	74	1378±18	70 (96)	31 (42)	3/11	13 (18)	37 (50)	5/19	72 (97)	3.7±0.3
With diabetes	≤65	15	1373±41	14 (93)	3 (20)	0/1	0 (0)	4 (27)	3/3	13 (87)	2.6±0.8^3,4,5^
	66–76	28	1358±36	25 (89)	11 (39)	1/2	8 (29)	9 (32)	3/11	27 (96)	5.5±1.1^3,4^
	≥77	31	1326±29	31 (100)	17 (55)	5/3	7 (23)	13 (42)	6/10	31 (100)	5.0±0.6^3,5^
	48–87	74	1348±20	70 (94)	31 (42)	6/6	15 (20)	26 (35)	12/24	71 (96)	4.7±0.5
All subjects	≤65	31	1424±29^2^	26 (84)	5 (16)	1/1	1 (3)	9 (29)	5/6	28 (90)	2.9±0.5
	66–76	54	1338±22^2^	51 (94)	23 (43)	2/6	11 (20)	22 (41)	5/17	52 (96)	4.6±0.6
	≥77	63	1354±19^2^	63 (100)	34 (54)	6/10	16 (25)	32 (51)	7/20	63 (100)	4.5±0.4
	48–87	148	1363±13	140 (95)	62 (42)	9/17	28 (19)	63 (43)	17/43	143 (97)	4.2±0.3

SE, standard error of means; *n*, number; HP*τ*, hyperphosphorylated tau; Aβ, amyloid-β; CAA, cerebral amyloid angiopathy; αS, α-synuclein; TDP43, transactive DNA binding protein 43, IR, immunoreactivity, ^a^number of IR patches of IAPP in ten randomly assessed fields in pancreas in magnification×100; ^b^a male subject, age 73 years at death with Multiple System Atrophy; ^c^a male subject, age 79 years at death with Progressive Supranuclear Palsy; ^d^a 50-year-old and ^e^an 80-year-old male with sparse HP*τ* pathology in hippocampus not sufficient for Braak staging[2]. Statistical differences, Kruskall-Wallis test ^1,2^*p* < 0.05, ^3^*p* < 0.005; Mann-Whitney U test ^4^*p* < 0.05, ^5^*p* < 0.01, ^6^*p* < 0.005.

### Brain weight

The brain weight decreased with age and differed significantly between the age groups (*p* < 0.05) in the whole cohort. This significant decrease was also noted for non-diabetics but lost in DM. A significant difference was noted between the youngest and middle aged subjects (*p* < 0.005) but only in non-diabetics (Table 2).

### The incidence of tissue alterations

The incidence of protein alterations increased with age in the whole cohort of 148 subjects (Table 2). HP*τ*/IR increased from 84 to 100%, Aβ/IR from 16 to 54%, αS/IR from 3 to 25%, pTDP43/IR from 29 to 51%, and IAPP/IR from 90 to 100%. Only 3% of the subjects lacked any IAPP/IR in their pancreas, and a high extent of IAPP/IR was observed in 8% of the subjects. The incidence of protein alterations was not influenced by DM. The incidence of vascular lesions both micro- and macroscopic seemed to increase with age and infarcts were more common in subjects with DM, 49% versus 32% (Table 2).

### The extent of altered proteins in the brainand pancreas and DM

The extent of HP*τ*/IR in the brain assessed applying the regional distribution of the misfolded protein increased with age. All of the youngest subjects (≤65 years) were in Braak stages 0–II, whereas most of the older subjects (≥66) were in Braak stages II–IV (Table 3). DM did not influence the extent of HP*τ*/IR.

**Table 3:** Percent of subjects with a given extent of hyperphosphorylated *τ* pathology (in three subjects * with hyperphosphorylated *τ* pathology, Braak staging could not be carried out)

Age group		Number	Braak stage [2]			
			0,a,b	I–II	III–IV	V–VI
≤65 years	all	30*	57	43
	non-diabetic	15*	80	20
	diabetic	15	33	67
66–76 years	all	54	37	41	13	9
	non-diabetic	26	23	46	23	8
	diabetic	28	50	35	4	11
≥77 years	all	61*	16	49	22	13
	non-diabetic	30*	13	50	17	20
	diabetic	31	19	48	26	7
All	all	145*	32	45	14	9
	non-diabetic	71*	31	42	16	11
	diabetic	74	34	47	12	7

The extent of Aβ/IR in the brain assessed applying the regional distribution of the misfolded protein increased with age. Most of the youngest subjects (≤65 years) did not display any Aβ/IR in the brain tissue, whereas phase 4–5 was primarily seen in the oldest subjects (≥76). DM did not influence the extent of Aβ/IR (Table 4).

**Table 4:** Percent of subjects with a given extent of amyloid-β pathology

Age group		Number	Thal phase				
			[7, 23]				
			0	1	3	4	5
≤65 years	all	31	87	13
	non-diabetic	16	94	6
	diabetic	15	80	20
66–76 years	all	54	61	22	4	7	6
	non-diabetic	26	54	23	4	8	12
	diabetic	28	68	21	4	7
≥77 years	all	63	46	29	6	13	6
	non-diabetic	32	47	22	9	16	6
	diabetic	31	45	36	3	10	6
All	all	148	60	23	4	8	5
	non-diabetic	74	59	20	5	9	7
	diabetic	74	61	26	3	7	3

The extent of αS/IR in the brain assessed applying the regional distribution of the misfolded protein increased with age. αS/IR was seen in 18% of the subjects, and most of these subjects were older than 65 years. In 8% of the whole cohort the highest stages of αS pathology (BNE stage 5 and 6) was seen. DM did not influence the extent of αS/IR (Table 5).

**Table 5:** Percent of subjects with a given extent of α synuclein pathology; One subject * with Multiple System Atrophy

Age group		Number	BNE stage [6]					
			0	1	3	4	5	6
≤65 years	all	31	97	3
	non-diabetic	16	94	6
	diabetic	15	100
66–76 years	all	53*	81	5	4	2	4	4
	non-diabetic	26	88	4			4	4
	diabetic	27*	74	7	7	4	4	4
≥77 years	all	63	76	3	5	5	8	3
	non-diabetic	32	72	3	3	6	13	3
	diabetic	31	81	3	7	3	3	3
All	all	148	82	4	3	3	5	3
	non-diabetic	74	82	4	1	3	7	3
	diabetic	73*	82	4	5	3	3	3

The extent of pTDP43/IR in the brain while assessing three commonly affected regions (amygdala, hippocampus, and medulla) increased with age. In 43% of the whole cohort pTDP43/IR was observed. In the youngest age group, 29% of subjects were affected with pTDP43 pathology and in 8% of the whole cohort all three assessed regions were affected. DM did not influence the extent of pTDP43/IR (Table 6).

**Table 6:** Percent of subjects with a given extent of transactive DNA-binding protein 43 (TDP43)

Age group		Number	TDP43 seen in 0, 1, any 2, all 3 regions			
			(medulla, amygdala, hippocampus)			
			0	1	2	3
≤65 years	all	31	71	26		3
	non-diabetic	16	69	31
	diabetic	15	73	20	7
66–76 years	all	54	59	28	7	6
	non-diabetic	26	50	34	12	4
	diabetic	28	68	21	4	7
≥77 years	all	63	49	21	17	13
	non-diabetic	32	41	19	18	22
	diabetic	31	58	23	16	3
All	all	148	57	25	10	8
	non-diabetic	74	50	27	12	11
	diabetic	74	65	22	8	5

The extent of IAPP/IR in pancreas while applying a semiquantitative assessment strategy increased with age when all subjects were included (Table 2). The increase was close to significant (*p* = 0.07) for the whole cohort but significant when comparing the youngest subjects with oldest (*p* < 0.05). When only diabetics were included the increase in the extent of IAPP/IR with age was significant (*p* = 0.021) and when the youngest group was compared with the middle-aged or the oldest group (*p* < 0.05 and *p* < 0.01 respectively). The mean extent of IAPP/IR did not differ significantly between DM and non-diabetic. In 57% of the subjects, the mean number of IAPP/IR patches was 4 or less and in 8% the mean number was more than 10 (Table 7). Fifteen percent of the subjects with DM had a high extent of IAPP/IR in their pancreas (IAPP/IR >10.1) when compared with 1% of the non-diabetic subjects. This difference was noted also for different age groups, i.e., 0 versus 7%, 0 versus 25%, and 3 versus 10% respectively.

**Table 7:** Percent of subjects with a given extent of amylin/islet amyloid polypeptide (IAPP)

Age group		Number	IAPP immunoreactivity in the pancreas					
			0	≤2.0	2.1–4.0	4.1–6.0	6.1–10.0	>10.1
≤65 years	all	31	10	35	26	13	13	3
	non-diabetic	16	6	31	25	19	19
	diabetic	15	13	40	26	7	7	7
66–76 years	all	54	4	30	26	15	12	13
	non-diabetic	26	4	31	23	23	19
	diabetic	28	4	28	29	7	7	25
≥77 years	all	63		29	25	19	21	6
	non-diabetic	32		38	25	12	22	3
	diabetic	31		19	26	26	19	10
All	all	148	3	31	26	16	16	8
	non-diabetic	74	3	34	25	17	20	1
	diabetic	74	4	27	27	15	12	15

### Correlation between altered proteins, the age of the subject and diabetes

Overall the highest correlations were observed between misfolded proteins seen in the brain (Table 8). High and significant correlation was observed in the 148 subjects between the age of the subjects and HP*τ*/IR, age and Aβ/IR, HP*τ* and Aβ/IR, HP*τ* and pTDP43/IR, Aβ and pTDP43/IR and Aβ and αS/IR. This association was preserved in the 74 non-diabetic subjects, whereas it decreased or was lost, i.e., was insignificant in subjects with DM. Extent of IAPP/IR showed weak but significant correlation with age in the 148 assessed subjects. This significant correlation was also observed for the subjects with DM. A negative correlation (*r* = –0.3, *p* < 0.05) was observed between IAPP/IR and Aβ/IR in cognitively unimpaired subjects without DM. A significant correlation was detected between IAPP/IR and pTDP43/IR in subjects with DM (*r* = 0.2, *p* < 0.05) and when only the 12 subjects with DM and that displayed extensive IAPP/IR (>9) were included the significance became higher (*r* = 0.8, *p* < 0.005). A significant correlation was also observed in cognitively unimpaired subjects with DM (*r* = 0.3, *p* < 0.05). This significant correlation was lacking in demented subjects with DM and was negative (*r* = –0.6, *p* < 0.05) in demented subjects without DM.

**Table 8:** Correlation between the extent of “proteinopathy” seen in the brain (HP*τ*, Aβ, αS and pTDP43) and in the pancreas (IAPP)

	Spearman correlation coefficient r															
		**age/**					**HP*τ*/**			**Aβ/**		**pTDP43/**	**IAPP/**			
	*n*	HP*τ*	Aβ	pTDP43	αS	IAPP	Aβ	pTDP43	αS	pTDP43	αS	αS	HP*τ*	Aβ	pTDP43	αS
All subjects	148	**0.4^**3**^**	**0.3^**3**^**	0.2^2^	0.2^1^	0.2^1^	**0.5^**3**^**	**0.4^**3**^**	0.2^2^	**0.3^**3**^**	**0.3^**3**^**	0.2^1^
nonDM	74	**0.5^**3**^**	0.3^2^	0.4^2^	0.2^2^		**0.7^**3**^**	**0.5^**3**^**	0.4^2^	**0.5^**3**^**	0.4^2^	0.3^1^
DM	74	0.3^1^	0.3^2^	0.3^1^			0.4^2^	0.3^2^							0.2^1^
nonCI	124	**0.4^**3**^**	0.3^2^	0.2^1^	0.2^2^		**0.3^**3**^**	0.3^2^		0.2^1^	0.2^1^
CI	24						**0.8^**3**^**
nonDM/nonCI	60	**0.5^**3**^**			0.3^1^		**0.5^**3**^**	0.3^1^	0.4^2^	0.3^1^	0.4^2^	0.3^2^		–0.3^2^
nonDM/CI	14						**0.9^**3**^**								–0.6^1^
DM/nonCI	64	0.3^1^	0.3^1^					0.3^1^							0.3^1^
DM/CI	10						0.9^2^

HP*τ*, hyperphosphorylated tau (extent Braak stage [2]); Aβ, amyloid-β (extent Thal phase [7, 23]); pTDP43, phosphorylated transactive DNA binding protein (extent: immunoreactivity to be seen in one, two or three regions, i.e., regions medulla, amygdala, hippocampus); αS, α synuclein (extent BNE stage [6]) IAPP, islet amyloid polypeptide (extent: the mean number of immunoreactive patches in ten randomly selected fields); DM, subjects with diabetes; nonDM, subjects without diabetes; CI, subjects with cognitive impairment; nonCI, subjects without cognitive impairment. Spearman correlation test ^1^*p* < 0.05, ^2^*p* < 0.01, ^3^*p* < 0.0001, *r* values when *p* < 0.0001 are given in bold.

### Dementia and diabetes

There were 24 subjects that had displayed cognitive impairment during life, 10 of them were subjects with DM (Table 9, cases 15 to 24). The most common cause of death was cardiovascular complication followed by bronchopneumonia, and DM did not influence the cause of death. The mean age at death (not significant) and the brain weight (*p* < 0.05) were lower in the group of cognitively impaired with DM when compared to the non-diabetics, and the mean extent of IAPP/IR (not significant) was higher in subjects with DM when compared with the non-diabetic group. The extent of all pathologies (HP*τ*, Aβ, αS, pTDP43) was higher (not significant) in subjects without diabetes. In both the DM and in the non-diabetic group some subjects displayed sparse and some extensive IAPP/IR. There were no significant differences in the definite diagnosis of neurodegeneration between subjects with or without DM (Table 9).

**Table 9:** Proteinopathy seen in the brain and pancreas in 24 subjects with cognitive impairment; case 1 to 14 without clinical diagnosis of diabetes, case 15 to 24 subjects with clinical diagnosis of diabetes

	IAPP^a^	Age	Sex	BW	Braak	Thal A*β*	CAA	BNE *α*S	pTDP43^b^	Vascular	PAD	NIA-AA
	mean±SE		M/F	grams	HP*τ*	phase	type [24]	stage [6]		pathology^c^		[52, 53]
					stage [2]	[7, 23]
1	1.2	72	M	1390	5	5	2	0	3	0	AD/TDP	High
2	8.7	75	F	1275	5	5	1	0	3	1	AD/TDP	High
3	3.4	75	M	1495	3	1	2	6	3	0	DLBD/TDP	intermediate
4	15.2	79	M	1515	0	0	0	0	0	0	PSP
5	5.1	79	M	1600	5	5	2	5	2	1	AD/LBD/TDP	High
6	2.3	80	F	1380	4	1	0	0	2	1	AD/TDP	intermediate
7	6.6	80	M	1413	5	4	0	6	0	0	AD/LBD	High
8	5.3	81	M	1215	5	4	2	0	2	0	AD/TDP	High
9	1.6	82	M	1370	2	0	0	0	3	0	FTLD/TDP
10	1.5	83	F	1580	5	4	2	0	3	0	AD/TDP	High
11	9.0	84	M	1493	5	3	0	4	3	0	AD/LBD/TDP	High
12	6.9	85	F	1310	1	0	2	0	0	0	HS
13	5.0	87	F	1160	5	5	2	0	3	0	AD/TDP	High
14	5.1	87	M	1215	2	1	0	0	3	0	FTLD/TDP
***n = 14***	**5.5** **±** **1.0**	**80.6** **±** **1.2**	**9/5**	**1387** **±** **37**^1^	5.0±0.4	2.7±0.6		1.5±0.7	2.0±0.3
15	4.2	62	F	1235	1	0	0	0	3	0	FTLD/TDP
16	1.5	69	F	1230	5	3	0	3	2	0	AD/LBD/TDP	High
17	3.7	69	F	1300	5	4	2	6	3	0	AD/LBD/TDP	High
18	1.2	73	M	1475	0	1	2	0	0	0	MSA
19	24.1	73	M	1300	6	4	0	0	1	0	AD/TDP	High
20	0.9	75	M	1365	3	0	0	0	0	1	AgD
21	10.4	81	F	1310	4	4	1	3	0	0	AD/LBD	intermediate
22	5.3	83	F	1060	3	3	0	0	0	1	AD	intermediate
23	7.3	84	F	988	5	5	2	0	2	0	AD/TDP	High
24	8.0	84	M	1290	2	0	0	0	0	0	FTLD/FUS	Low
***n = 10***	**6.7** **±** **2.2**	**75.3** **±** **2.4**	**4/6**	**1255** **±** **45**^1^	4.3±0.7	2.1±0.7		1.3±0.7	1.1±0.4
***n = 24***	**6.0** **±** **1.1**	**78.4** **±** **1.3**	**13/11**	**1332** **±** **31**	4.7±0.4	2.5±0.4		1.4±0.5	1.6±0.3

IAPP, islet amyloid polypeptide; ^a^mean number of IAPP immunoreactive patches in ten randomly selected fields assessed in magnification x100: n, number; M, male; F, female; BW, brain weight; HP*τ*, hyperphosphorylated tau; A*β*, *β* amyloid; CAA, cerebral amyloid angiopathy; *α*S, *α* synuclein; pTDP43, phosphorylated transactive DNA binding protein; ^b^pTDP43 immunoreactivity to be seen in 1, 2, or 3 regions (medulla, amygdala, hippocampus);^c^macro- and/or microscopic lesions observed in hematoxylin-eosin stain in any of the assessed section; PAD, PathoAnatomical Diagnosis; AD, Alzheimer’s disease; AgD, Argyrophilic grain Disease; DLBD, Diffuse Lewy body dementia; FTLD, Frontotemporal Lobar degeneration, FUS, fused in sarcoma; HS, hippocampal sclerosis; LBD, Lewy body disease; MSA, Multi System atrophy; NIA-AA, National Institute on Aging– Alzheimer’s Association; Significant difference, non-parametric Mann-Whitney-U test^1^ *p* < 0.05.

### Colocalization of Aβ and IAPP

IAPP/IR was not observed in the 28 sections of the frontal cortex with a high load of Aβ/IR, and Aβ/IR was not observed in the 15 pancreas sections with a high load of IAPP/IR.

## DISCUSSION

Here we found that the incidence and the extent of age related misfolded proteins seen in the brain or the pancreas increased with age. Noteworthy, this increase was not influenced by DM. Thirteen percent of our autopsy cohort of 615 subjects, age ranging from 48 to 102 years had DM, based on the medical records. In 93% of subjects with DM, tissue was available for this study. The percentage of 13% diabetics is well in line with the reported incidence of 10 to 20% of DM observed in Europeans over 65 years of age [25].

### IAPP immunoreactivity in pancreas in aged

Numerous reports have indicated that aggregation of IAPP in the pancreatic tissue is a phenomenon related to DM [10, 11]. In 1990, it was reported that altered IAPP aggregates were seen in the pancreas in up to 77% of DM when compared to 7% in non-diabetic subjects [26]. In this study, we assessed the IAPP/IR in the endocrine part of the pancreas (the islets of Langerhans) applying IHC techniques. In many of the cases, the tissue was altered due to the postmortem delay, but the IHC techniquereadily visualized the IAPP/IR. The youngest subject (48 years old) had DM and already displayed IAPP/IR in pancreas, whereas lack of IAPP/IR was registered in only five subjects, age ranging from 50 to 71 years. Thus, contrary to the report by Clark and colleagues, we observed that almost all our subjects (97%), independent of clinical diagnosis of DM, displayed IAPP/IR in their pancreas. The discrepant result when comparing our results with Clark and colleagues might be related to selection bias, methods used and to the population studied (Scandinavians versus Pima Indians). Contrary to our expectations, three of the subjects with DM lacked IAPP/IR and this might be due to selection bias; only one section obtained from the body of pancreas was investigated. Surprisingly, almost all aged non-diabetics in our study displayed IAPP/IR and this might be explained by the recent observations that a large number of adults in the U.S. have undiagnosed type 2 DM, and 5 to 10% of the European population display impaired glucose regulation [27, 28].

Not previously reported to our knowledge, we observed that the extent of IAPP/IR increased with age, and this increase was noted for the whole cohort of 148 subjects and for the 74 subjects with DM. Thus the increase of extent of IAPP/IR in the pancreas is an age- and DM-related phenomenon, i.e., IAPP/IR was significantly higher in the oldest group when compared to the youngest. This observation is in line with the previous reports indicating that the prevalence of impaired glucose regulation seems to increase with age [28]. The mean number of labeled islets of Langerhans in the pancreas did not differ significantly between the subjects with DM and non-diabetics, but a high extent of IAPP/IR was observed more often in subjects with DM. The reports indicating that there is a substantial number of undiagnosed subjects with type 2 DM or with impaired glucose regulation is intriguing [27, 28]. The question arises as to whether the pathology seen by us postmortem is a sign of a prodromal stage of DM.

### Misfolded proteins in the brain

The prevalence of aggregation prone proteins in the brain, i.e., HP*τ*, Aβ, α-S, and pTDP43, increased with age. The most common alteration to be observed was HP*τ* followed by Aβ, pTDP43, and αS. Thus, our results here are in agreement with previous reports [1]. This prevalence did not seem to be significantly influenced by DM. It was not only the prevalence but also the extent of HP*τ*, Aβ, α-S, and pTDP43 that increased with age. The Braak stage of HP*τ*, the Thal phase of Aβ, and the BNE stage of αS increased with age for the whole study population. When assessed separately for DM and non-diabetics, no major differences in the patterns of the age-related changes were seen. These results are in line with previous reports [20]. A similar trend was also observed for pTDP43 pathology as assessed here, i.e., number of brain regions affected. Thus, our results imply that DM does not influence the prevalence or the extent of altered proteins to be seen in the aging brain.

In addition to altered proteins, the prevalence of vascular lesions increased with age in both DM and non-diabetic subjects, and vascular lesions were found to be more frequent in DM. This observation is in line with previous reports indicating that an association exists between the extent of vascular lesions and DM [29].

### Correlation between the misfolded proteins

Extent of all assessed misfolded proteins was significantly influenced by the age. A significant correlation was also observed between HP*τ*, Aβ, pTDP43, and αS. Thus an association between AD related misfolded proteins and pTDP43 and αS/IR was observed. This might suggest that the AD related aggregation prone proteins might act as a seed to initiate aggregation of other proteins and thus this hypothesis should not be overlooked [18]. Contrary to the above, no correlation was observed between HP*τ*, Aβ, and αS/IR in the brain and IAPP/IR in the pancreas. The extent of IAPP/IR did not show any association with AD-related pathology; moreover, a negative correlation was observed between IAPP and Aβ/IR in cognitively unimpaired non-diabetics. Therefore, our results obtained in humans at the end stage, i.e., postmortem, do not support the epidemiological, clinical, or experimental data suggesting that a causative relationship exists between DM and AD, i.e., misfolded protein IAPP acts as a seed for misfolding of Aβ, HP*τ*, and αS or vice versa [19]. Interestingly the extent of pTDP43/IR seen in as many as 43% of our cohort was significantly associated with IAPP/IR in subjects with DM. Noteworthy, when only 12 subjects with both DM and extensive IAPP/IR were included the correlation was strong (*r* = 0.8) and significant (*p* < 0.005). This correlation was also noted in the 64 cognitively unimpaired subjects with DM but was lacking in the 10 demented with DM. Surprisingly, a significant negative correlation between pTDP43 and IAPP/IR was observed for the 14 non-diabetic demented subjects (Table 8). Therefore, based on our finding, pTDP43/IR seems to be associated with IAPP/IR and DM. Recently it was reported that there is an interaction between IAPP and a single nucleotide polymorphism (SNP) on chromosome 12p12 (rs73069071) and this SNP has been shown to be associated with hippocampal sclerosis (HS) [30, 31]. Parallel with this, an association between HS and pTDP43 has been proposed by many [32, 33]. Furthermore, in TDP43 transgenic mice impaired insulin mediated glucose uptake has been described [34]. Thus, there is indeed evidence for interplay between IAPP and TDP43. TDP43 is ubiquitously expressed in all organs, binds to both DNA and RNA and has multiple functions including transcriptional repression, pre-mRNA splicing and translational regulation [35, 36]. Whether phosphorylation of TDP43 is influenced by impaired glucose metabolism has not yet been studied and whether phosphorylation of TDP43 can be observed in peripheral organs in diabetes has not been looked for. It is reported that up to 50% of AD patient display concomitant pTDP43 pathology and whether subjects with AD and concomitant pTDP43 suffer from DM has not been looked for [37].

### Colocalization of Aβ and IAPP

Already in 2010, it was reported that AD-related pathology was seen in pancreas in diabetics [38]. Following in 2013, it was reported that IAPP/IR was observed in the brains of demented subjects with type 2 DM [39]. We carried out the IHC stain on those cases that displayed an excess of the primary pathology (Aβ or IAPP) and applied mAb while staining for IAPP and Aβ. We were unable to repeat any of the reported observations given above. There was no Aβ/IR seen in the pancreas in 15 subjects with excess of IAPP/IR. Twelve of these subjects had DM, and 3 of these subjects had displayed cognitive impairment. There was no IAPP/IR seen in the 28 brain samples with excess of Aβ/IR. Twelve of these subjects had DM and 13 were demented. The differing results are probably due to the methodology, both tissue and methodology related. Recently in 2017, cross-seeding and colocalization of IAPP and Aβ was reported to be seen in transgenic mice [19]. This finding was further highlighted by Ridler [40]. One factor that should be considered in the described experimental setting by Moreno-Gonzales and colleagues is the physiological increase of amyloid-β protein precursor (AβPP) production seen in posttraumatic brains [41]. Thus, the inoculation of pancreatic aggregates into the brain (a trauma) as was carried out by Moreno-Gonzales and colleagues might cause a physiological increase in AβPP production that in a setting of AβPP transgenic mice might lead to Aβ aggregation.

### Dementia and diabetes

The overall incidence of dementia in developed countries has been reported as decreasing during the last few years, from 11.6% to 8.8% [42]. This decrease is considered as being attributed to social, behavioral, and medical factors. Out of our 148 subjects, 16% of the subjects displayed cognitive impairment. This incidence is somewhat higher than overall incidence probably due to a selection bias, i.e., reason to refer for autopsy varies. The distribution of dementia in our study cohort with or without DM was fairly even (10 and 14 subjects, respectively). There was no notable difference in the distribution of the definite diagnosis of dementia. One obstacle, however, is the lack of consensus criteria regarding vascular lesions to be assessed as being causative for dementia thus the vascular pathology might have been overlooked [43].

A link between cognitive impairment, i.e., dementia and DM has been suggested in epidemiological and clinical studies and in particular, it has been proposed that there is an association between DM and AD [17, 44, 45]. This presumption has been challenged by others suggesting that dementia in DM is primarily related to vascular alterations [20, 29, 46–48]. Here, based on our results, assessing the hallmark lesions of AD and other dementias and the alteration related to DM, i.e., IAPP/IR, no association could be seen between DM and AD. The lack of a link between AD and DM is also supported by the observations that less AD related pathology has been reported to be seen in subjects with DM when compared to non-diabetic subjects [21, 49, 50]. Our results are in line with the above, as our demented subjects with DM displayed less misfolded proteins (not significant) in their brain when compared with non-diabetics. Further, in 2015, Moran and colleagues reported that no association could be found between DM and Aβ in the brain when assessed with ^11^C Pittsburgh compound B or with cerebrospinal fluid Aβ_42_ levels [22]. In line with the above, Kuo and colleagues reported in 2015 that the hazard ratio for dementia in DM was associated with comorbidities such as hypertension, hyperlipidemia, coronary artery, and/or kidney disease— all risk factors for vascular lesions in the brain [51]. In line with the above vascular alterations were seen in 49% of subjects with DM when compared with 32% in non-diabetics in our cohort.

### Strength and weaknesses of the study

The strength of this study is that we have assessed the pathological alterations in the brain and in the pancreas that have been reported as being associated with clinical conditions such as DM and dementia. The selection of cases was based on a known clinical condition, i.e., DM, the prevalence being in line with what is seen in the general population. An age and gender matched control group was selected from the original autopsy cohort. The prevalence of dementia was quite evenly distributed in non-DM and DM. We were able to assess objectively both the incidence and the extent of neurodegenerative alterations as well as the DM related aggregation of IAPP seen in the pancreas. One of the major weaknesses of the study is the assessment of vascular lesions limited to infarcts and cerebral amyloid angiopathy and the lack of consensus of vascular pathology to be assessed in the postmortem brain of demented. Thus, the vascular pathology might have been overlooked.

### Conclusion

We found that the incidence and the extent of HP*τ*, Aβ, pTDP43, αS, and IAPP increased with age, independent of DM. The definite diagnosis of dementia varied in both the DM and the non-diabetic cohort. We noted that two of the misfolded proteins were observed in virtually all of the subjects, i.e., HP*τ* in the brain and IAPP in the pancreas. The high incidence of HP*τ* pathology in the brains of the aged is well known, whereas the observation of IAPP in the pancreas in almost all of the subjects, even those not clinically diagnosed with DM, is intriguing. This might be related to a high number of aged displaying impaired glucose regulation and/or undiagnosed type 2 DM, as recently reported. A strong correlation was observed between HP*τ*, Aβ, αS, and pTDP43 that is in line with the cross-seeding hypothesis, whereas lack of correlation between HP*τ*, Aβ, αS, and IAPP argues against this molecular mechanism in development of these lesions. Therefore, we are unable to confirm the suggested link between AD and DM. Interestingly an association was observed between pTDP43 and IAPP/IR, not previously reported. The association IAPP/pTDP43 was strong and significant particularly in subjects with DM and high extent of IAPP/IR in pancreas. A link between IAPP/HS, TDP43/HS, and TDP43/glucose homeostasis have been recently suggested indicating that an association between these two protein alterations might be of significance [30–34]. TDP43 is ubiquitously expressed in all organs and whether phosphorylation of TDP43 is influenced by impaired glucose metabolism or pTDP43 is observed in peripheral organs in diabetes has not yet been studied. The reported association between AD and DM (epidemiological and clinical studies) might, based on our results, be related to the high incidence of pTDP43 seen postmortem in AD patients. Thus, pTDP43 should urgently be looked for in other organs in diabetics and in experimentalmodels and the influence of glucose metabolism on phosphorylation of TDP43 should urgently be explored.
